# Genetic diversity and population structure studies of West African sweetpotato [*Ipomoea batatas* (L.) Lam] collection using DArTseq

**DOI:** 10.1371/journal.pone.0312384

**Published:** 2025-01-03

**Authors:** Issa Zakari Mahaman Mourtala, Arnaud Comlan Gouda, Dan-jimo Baina, Nwankwo Innocent Ifeanyi Maxwell, Charlotte O. A. Adje, Moussa Baragé, Oselebe Ogba Happiness

**Affiliations:** 1 Department of Natural Resources Management, National Institute of Agronomic Research of Niger, Niamey, Niger; 2 Africa Rice Center, M’bé Research Station, Bouaké 01, Côte d’Ivoire; 3 Sweetpotato Research Programme, National Root Crops Research Institute, Umudike, Abia State, Nigeria; 4 Genetics, Biotechnology and Seed Science Unit (GBioS), Laboratory of Crop Production, Physiology and Plant Breeding (PAGEV), Faculty of Agricultural Sciences, University of Abomey-Calavi, Cotonou, Benin; 5 Faculty of Agronomy, Abdou Moumouni University of Niamey, Niamey, Niger; 6 Department of Crop Production and Landscape Management, Ebonyi State University, Abakaliki, Nigeria; KGUT: Graduate University of Advanced Technology, ISLAMIC REPUBLIC OF IRAN

## Abstract

**Background:**

Sweetpotato is a vegetatively propagated crop cultivated worldwide, predominantly in developing countries, valued for its adaptability, short growth cycle, and high productivity per unit land area. In most sub-Saharan African (SSA) countries, it is widely grown by smallholder farmers. Niger, Nigeria, and Benin have a huge diversity of sweetpotato accessions whose potential has not fully been explored to date. Diversity Arrays Technology (DArTseq), a Genotyping by Sequencing (GBS) method, has been developed and enables genotyping with high-density single nucleotide polymorphisms (SNPs) in different crop species. The aim of this study was to assess the genetic diversity and population structure of the West African sweetpotato collection using Diversity Arrays Technology through Genotyping by Sequencing (GBS).

**Results:**

29,523 Diversity Arrays Technology (DArTseq) single nucleotide polymorphism markers were used to genotype 271 sweetpotato accessions. Genetic diversity analysis revealed an average polymorphic information content (PIC) value of 0.39, a minor allele frequency of 0.26, and an observed heterozygosity of 10%. The highest value of polymorphic information content (PIC) (0.41) was observed in chromosomes 4, while the highest proportion of heterozygous (He) (0.18) was observed in chromosomes 11. Molecular diversity revealed high values of polymorphic sites (Ps), theta (θ), and nucleotide diversity (π) with 0.973, 0.158, and 0.086, respectively, which indicated high genetic variation. The pairs of genetic distances revealed a range from 0.08 to 0.47 with an overall average of 0.34. Population structure analysis divided the 271 accessions into four populations (population 1 was characterised by a mixture of accessions from all countries; population 2, mostly comprised of Nigerian breeding lines; population 3 contained exclusively landraces from Benin; and population 4 was composed by only landraces from West African countries) at K = 4, and analysis of molecular variance (AMOVA) based on *PhiPT* values showed that most of the variation was explained when accessions were categorized based on population structure at K = 4 (25.25%) and based on cluster analysis (19.43%). Genetic distance showed that group 4 (which constituted by landraces of Niger and Benin) was genetically distant (0.428) from groups 2 (formed by 75% of breeding lines of Nigeria), while group 1 was the closest (0.182) to group 2.

**Conclusions:**

This study employed 7,591 DArTseq-based SNP markers, revealing extensive polymorphism and variation within and between populations. Variability among countries of origin (11.42%) exceeded that based on biological status (9.13%) and storage root flesh colour (7.90%), emphasizing the impact of migration on genetic diversity. Population structure analysis using principal component analysis (PCA), Neighbor-Joining (NJ) tree, and STRUCTURE at K = 4 grouped 271 accessions into distinct clusters, irrespective of their geographic origins, indicating widespread genetic exchange. Group 4, dominated by landraces (95%), showed significant genetic differentiation (Nei’s Gst = 0.428) from Group 2, mainly comprising breeding lines, suggesting their potential as heterotic groups for breeding initiatives like HEBS or ABS.

## Introduction

Sweetpotato [*Ipomoea batatas* (L.) Lam] is a dicotyledonous angiosperm plant that belongs to the *Convolvulaceae* family [1]. It is a hexaploid species (2n = 6x = 90) [2], with two non-homologous genomes (B1B1B2B2B2B2) and tetradisomic inheritance [1]. Sweetpotato originated in Central America, where it was found growing in the wild and then spread across the Pacific from Central America [3, 4]. It was later transported to warmer regions of Asia and Africa by Spanish and Portuguese traders [2, 5]. It was probably first introduced as a root crop in Tanzania, and then the crop dispersed from East to West Africa [1]. In terms of nutritional value, sweetpotato crops contain high levels of beta-carotene, anthocyanins, phenolics, dietary fibre, vitamins, minerals, and other bioactive compounds [6, 7]. Compositional analysis has shown that orange-fleshed sweetpotato varieties are a superior source of β-carotene [8], while purple-fleshed sweetpotato varieties have excellent levels of anthocyanins [9]. In contrast, white-fleshed storage root sweetpotato varieties contain little to no β-carotene [8].

Sweetpotatoes have special attributes such as adaptability to a wider topography, the ability to grow in subsidiary circumstances, good productivity in short durations, and a balanced nutritional composition [10]. Asia, being the largest producer of sweetpotato in the world, (61.5%), followed by Africa (33.7%) [11]. With a compound annual growth rate of 3.3% in area and 5.1% in production, sweetpotato production has increased in sub-Saharan Africa (SSA) over the past 20 years (1997–2016) [12]. In West Africa, the crop is gaining importance. For instance, in 2022, Nigeria is the largest producer of sweetpotato in the region with annual production of (3,943,046 tons), Niger, came fifth (224,017 tons), while Benin, came in tenth (53,894 tons), positionamong the top ten sweetpotato producer in West Africa [11]. Sweetpotato yield levels can vary considerably worldwide due to soil mineral compositions, weather, and crop variety. Under ideal conditions, very high yields can be attained. However, productivity and quality are often limited by abiotic and biotic stresses. Sweetpotatoes exhibit significant agro-morphological and physico-chemical variability that can be used to counteract stress-related production losses [13].

Sub-Saharan Africa is regarded as a secondary center of diversity of sweetpotato after tropical America [14], but genetic characterization of sweetpotato varieties from the region is limited. Countries like Niger, Nigeria, and Benin alone have a great diversity of sweetpotato accessions that have not yet been thoroughly explored for their potential. Additional barriers to sweetpotatoes exploitation, include several difficulties such as conventional breeding combine with its large and complex genomes (2n = 6x = 90) [15]. Previous research in other crops, including rice [16], has demonstrated, a better understanding of sweetpotato genetic variability in SSA is crucial for selection of parental genotypes. According to [16] genetic diversity analysis of germplasm collection can be undertaken using agro-morphological traits, biochemical markers and molecular markers. Morphological markers have been used in studying the genetic diversity of sweetpotato [17, 18], but these markers are influenced by the environment, and are limited in number. Moreover, chemical assays of isozymes are also relatively few [19]. Molecular markers, on the other hand, accurately depict the genetic diversity between genotypes at the deoxyribonucleic acid (DNA) level [20]. Different types of molecular markers have been widely used in genetic analysis [19]. Simple Sequence Repeats (SSR) markers have been successfully used for sweetpotato genetic diversity analyses in West African countries of Burkina Faso [21], Ghana [13], Togo [22], and Nigeria [23]. Currently, SNP markers have become the reference type of DNA markers for plant breeding. Single Nucleotide Polymorphisms (SNPs) increase information of genome wide marker and has small missing marker, and it is also rapid [24, 25]. However, the exploration of West African sweetpotato genetic diversity and population structure using single nucleotide polymorphism (SNP) markers has not yet been reported.

Recently, Diversity Array Technology (DArT) has developed a Genotyping by Sequencing (GBS) method called “DArTseq” for genotyping with high-density SNPs. This technology is a relevant tool for different studies in genome applications. The advantage is that today it is applicable for large and complex genome species [26–28]. GBS has been applied to different species, including wheat [29], common bean [30], sesame, [20], rice [31, 32], taro [33], and sweetpotato [34–36], but it hasn’t been widely applied to the sweetpotato accessions from West Africa. In essence, despite the crop’s pivotal role in West Africa, the genetic characterization of its diverse accessions in the region has been limited. Therefore, the primary objective of this study was to enhance the characterization of the West African sweetpotato collection using Diversity Arrays Technology (DArT) through Genotyping by Sequencing (GBS). This study represents the inaugural comprehensive exploration of West African sweetpotato genetic diversity using DArTseq-based SNP markers. By leveraging this advanced genotyping technology, we have gained new insights into the genetic relationships and population structure of sweetpotato in the region. Thus, the overarching aim of this research was to refine the characterization of West African sweetpotato collection using Diversity Arrays Technology (DArT) through Genotyping by Sequencing (GBS). The specific objectives were: (*i*) to assess the genetic diversity of 271 sweetpotato clones using DArTseq SNP markers; (*ii*) to investigate the genetic relationships among the accessions and their geographic origin; and (*iii*) to delineate the population structure. This comprehensive genetic profiling will empower breeders to effectively harness the available sweetpotato diversity in West Africa for the development of improved cultivars.

## Materials and methods

### Plant materials

A total of 271 sweetpotato accessions were used in this study (S1 Table). These samples were collected as vine cuttings and storage roots from Niger farmers’ fields (4 accessions), Nigeria National Root Crop Research Institute, Umudike (140 accessions) and Abomey-Calavi University in Benin republic (127 accessions). The accessions included landraces, breeding lines, and improved varieties (S1 Table). Fresh leaf (about 3 weeks old) from each accession was collected and shipped to the Integrated Genotyping Service and Support (IGSS) platform at the Biosciences Eastern and Central Africa-International Livestock Research Institute (BecA-ILRI) Hub in Nairobi for genotyping.

### DNA extraction and library construction

Fresh leaf samples harvested were transferred to 1.1 mL MicroTubes (Bioquote Limited, UK), dried at 57°C using a Binder FD53 E2 Drying oven (Akribis Scientific Limited, UK), covered with micronic sealing mats (NBS Scientific, USA), and shipped to Diversity Arrays Technology (DArT) Pty Ltd (http://www.diversityarrays.com/) for analysis. Genomic deoxyribonucleic acid (DNA) was extracted using the NucleoMag Kit following the procedure described in the NucleoMag Tissue User manual (www.takarabio.com). The extracted deoxyribonucleic acid (DNA) had a concentration of 50-100ng/ul. The quality and quantity of deoxyribonucleic acid (DNA) samples were evaluated by running deoxyribonucleic acid (DNA) on 0.8% agarose and using a spectrophotometer respectively. Libraries were constructed according to Kilian et al [37]. Briefly, the Diversity Arrays Technology (DArTSeq) complexity reduction method was used to construct libraries through digestion of genomic deoxyribonucleic acid (DNA) and ligation of barcoded adapters, followed by Polymerase Chain Reaction (PCR) amplification of adapter-ligated fragments. Libraries were sequenced using Single Read sequencing runs for 77 bases using Hiseq 2500 [37].

### Genotyping using DArT SNP markers

We used Diversity Arrays Technology (DarTseqTM), which is accessible via Integrated Genotyping Service and Support (IGSS) platform (https://ordering.igss-africa.org/cgi-bin/order/login.pl). This technology allows quick, high-quality, and cost-effective genome profiling, even from the most complex polyploid genomes. Diversity Arrays Technology (DArTseq) markers were scored using DarTsoft14, an in-house marker scoring pipeline based on built-in algorithms. Single Nucleotide Polymorphism (SNP) markers were scored in binary format to represent the presence/absence (1 and 0, respectively) of the restriction fragment with the marker sequence in the samples’ genomic representation and letter data format [37, 38]. Diversity Arrays Technology Single Nucleotide Polymorphism (DArT SNP) markers were aligned to the reference genomes of wild sweetpotatoes *I Trifida* and *I Triloba* to identify their chromosome positions. In sum, each accession was genotyped with 29,523 Diversity Arrays Technology Single Nucleotide Polymorphism (DArT SNP) markers.

### Filtering of SNP markers

The DArTSeq SNPs were generated, containing 29,523 Diversity Arrays Technology Single Nucleotide Polymorphism (DArTseq SNP) markers that were polymorphic across the 271 accessions of sweetpotato. High-quality SNP markers were selected based on a data filtering procedure adopted by Gemenet and collaborators [36]. All Diversity Arrays Technology Single Nucleotide Polymorphisms (DArT SNPs) without a chromosome position and those on chromosome zero (Chr00) were removed. Then, the genotype dataset from chromosomes 1 to 15 were filtered based on (i) ≤ 25% missingness, (ii) ≥ 0.25 polymorphic information content (PIC), and (iii) ≥ 10% minor allele frequency (MAF). Finally, 7,591 Single Nucleotide Polymorphism (SNP) markers were retained and deemed appropriate for all diversity study parameters on the 271 accessions.

### Statistical analysis

Most of the statistical analyses were performed as described previously [32, 39].

Briefly, TASSEL v.5.2.58 [40] was used to compute heterozygosity (He), identify-by-state (IBS)-based genetic distance matrices, principal component analysis (PCA), and construct Neighbour-joining (NJ) trees. The first three principal components (PCs) from the PCA were plotted using *R* package rgl version 1.0.1 (https://cran.r-project.org/web/packages/rgl/rgl.pdf). The analysis of molecular variance (AMOVA) [41], gene flow (Nm) and gene diversity (GI) were performed using the R/poppr package version 2.9.3 [42] to detect the genetic variance within and among populations using the *PhiPT* value (an analogue of Fst fixation index) [43]. To do this, accessions were assigned to 3–7 groups (populations) based on their state of collection, country of origin, region in the African continent, storage root flesh colour, biological status, or group membership predicted from the phylogenetic and population structure analyses. Also, Shannon’s diversity index (I), of DArT SNPs markers were obtained through R/poppr package version 2.9.3 [42].

To construct the Neighbour-joining (NJ) trees, each data set was converted to the phylo “interleaved” format and imported in R v 4.0.3 using the ape package version 5.4–1 [44]. Using R/phangorn package version 2.5.5, Neighbour-joining (NJ) trees were built with a maximum likelihood approach [45]. The “Newick” format of the neighbour-joining (NJ) trees built was exported and further refined using iTOL v4 online program [46].

### Analysis of genetic relatedness and population structure

Population structure analysis, was performed by exporting the HapMap format of each dataset to PHYLIP interleaved format using TASSEL v.5.2.57, which was then converted to Molecular Evolutionary Genetics Analysis (MEGA) X [47] and STRUCTURE v.2.3.4 [48] formats using PGDSpider v.2.1.1.3 [49]. MEGA X software was used to compute the number of segregating sites (S), the proportion of polymorphic sites (Ps), Theta (θ), and nucleotide diversity (π). The cross-entropy criterion estimating for clusters choosing [50] was performed on R package LEA [51]. The number of ancestral populations (K) was set to 1–30, and each K was repeated 100 times. The Bayesian Information Criterion (BIC) was also utilized to evaluate the best-supported model, as well as the number and nature of clusters, using [52].

Population structure was analyzed using the model-based method implemented in the software STRUCTURE v.2.3.4 [48] as described previously [31, 32]. Deoxyribonucleic acid (DNA) samples and accessions with membership probabilities > 60% were assigned to the same clusters (group), whereas those with probabilities < 60% in any group were assigned to a “mixed” group.

## Results

### Genotyping of West African sweetpotato

Of the 29,523 DArTseq SNPs markers used for genotyping (as shown in Table 1), only 25.71% (7,591 SNPs) were found to be polymorphic and mapped onto the 15 chromosomes of the sweetpotato. The number of mapped SNPs varied from 344 on chromosome 8 to 773 on chromosome 4, with an overall average of 506 SNPs per chromosome. The physical length of each chromosome ranged from 19,014 kilobase (kb) on chromosome 14 to 32,291 kb on chromosome 4 (Table 1; S1 Fig), and the total physical length was 369,150 kb. The average map length per SNP ranged from 42 kb on chromosome 4 to 58 kb on chromosome 2 (Table 1; S1 Fig).

**Table 1:** The chromosomal distribution of 7,591 polymorphic Single Nucleotide Polymorphisms (SNPs) used for genotyping 271 *Ipomoea batatas* accessions, including the physical length of each chromosome covered by the SNPs (in kb pairs) and average map length per SNP (kb).

Chromosome	Physical length based on 7,591 SNPs (*kb)	Number of SNPs polymorphic in 271 *Ipomoea batatas*	Average map length per SNP (*kb)
1	32,144	665	48
2	27,278	469	58
3	28,337	555	51
4	32,291	773	42
5	25,539	484	53
6	25,108	478	53
7	23,731	541	44
8	19,099	344	56
9	23,327	524	45
10	24,441	494	49
11	19,080	372	51
12	24,115	513	47
13	22,685	461	49
14	19,014	450	42
15	22,961	468	49
**Total**	**369,150**	**7591**	**-**

SNP = Single Nucleotide Polymorphism, kb = kilobase

### Genetic diversity and distance

To evaluate the genetic diversity across all genotyped West African sweetpotato accessions, the Polymorphism Information Content (PIC), Minor Allele Frequency (MAF), and Proportion of Heterozygosity (He), as well as genetic distance, were computed separately. The Polymorphism Information Content (PIC) was used to evaluate the quality of each of the 7,591 polymorphic SNPs. The PIC value was rather high and ranged from 0.25 to 0.50, averaging 0.39. Thirteen DArTseq SNP markers had a PIC higher than the computed mean value (S2 Table). The minor allele frequency for the 7,591 SNPs ranging from 0.10 to 0.50 with an average of 0.26 (S2 Table). The observed heterozygosity per accession ranged from 5 to 18% with an average of 10% (S1 Table). The average genetic distance between any two accessions was 0.34, with a range of 0.08 to 0.47 (S3 Table). Most pairs of accessions (80.78%) had a genetic distance between 0.30 and 0.40. Among all possible pairs of accessions, only 9.43% and 3.36% had genetic distances that fell from 0.20 and 0.30, and 0.10 and 0.20, respectively. The remaining 6.41% of pairs of accessions had a genetic distance between 0.40 and 0.50 (S2 Fig and S3 Table). In all loci, the average Shannon diversity index (I) was 0.75 (S2 Table).

### Population differentiation and genetic structure

The analysis of molecular variance (AMOVA) was performed at seven different levels of categorisation, including state of collection, country of origin, regions in Africa, biological status, sweetpotato storage root flesh colour, cluster, and STRUCTURE (Table 2). The proportion of variation among populations calculated for all categories ranged from 5.57% (groups based on regions in Africa) to 25.25% (STRUCTURE at K = 4), while within-population variation ranged from 74.74% to 94.43%. The genetic variance partitioned based on *PhiPT* values (Table 2) indicated that most of the genetic diversity was explained by population structure at K = 4 (25.25%). All *PhiPT* values for the 271 accessions in the different categories considered were significant (P < 0.001).

**Table 2:** Analysis of molecular variance (AMOVA) for the extraction of SNP variation among and within groups (populations) based on 271 *Ipomoea batatas* accessions genotyped with 7,591 polymorphic SNPs into seven different categories.

Category	Source of variation	Df	Sum of squares	Variance components	Percentage of variation	*PhiPT*
State of collection*	Between populations	4	38,745,25	19.7577	11.41	
	Within populations	266	408,298,94	153.4958	88.59	
	Total	270	447,044,19	173.2536	100.0	0.1140
Country of origin*	Between populations	4	36,320,35	19.8963	11.42	-
	Within populations	266	410,723,84	154.4074	88.58	-
	Total	270	447,044,19	174.3038	100.0	0.1141
Regions in Africa continent*	Between populations	2	57,216,28	97.2275	5.57	-
	Within populations	268	4,413,225,64	164.6725	94.43	-
	Total	270	4,470,441,92	174.3953	100.0	0.0557
Flesh colour*	Between populations	5	3474.005	13.32985	7.90	-
	Within populations	265	41,230,414	155.58647	92.10	-
	Total	270	44,704,419	168.91632	100.0	0.0789
Biological status*	Between populations	2	25,469,08	15.8009	9.13	-
	Within populations	268	4,21,575,11	157.3041	90.87	-
	Total	270	447,044,19	173.1050	100.0	0.0912
Groups based on cluster analysis	Between populations	3	70,911,56	33.9745	19.43	-
	Within populations	267	376,132,63	140.8736	80.57	-
	Total	270	447,044,19	174.8481	100.0	0.1943
Groups based on STRUCTURE at K = 4	Between populations	4	96,874,39	444.7979	25.25	-
	Within populations	266	350,169,80	1316.4378	74.74	-
	Total	270	447,044,19	17.612.258	100.0	0.2525

**State of collection**: Abia, Ebonyi, Dosso, Tillaberi and Atlantique; **Country of origin**: Nigeria, Uganda, Mozambique, Niger and Benin; **Regions in Africa continent**: West Africa, East Africa and South Africa; **Flesh colour**: white, cream, yellow, orange, purple and unknown; **Biological status**: landraces, breeding lines and improved lines; **Groups based on cluster analysis**: 4 groups; **Groups based on STRUCTURE at K = 4**: group 1, group 2, group 3, group 4 and mixed

### Molecular diversity indices and genetic differentiation

The estimated values for polymorphic sites (Ps), theta (θ), and nucleotide diversity (π) after analysing all 271 accessions were 0.973, 0.158, and 0.086, respectively (S4 Table). The nucleotide diversity (π) computed between improved lines (19 accessions) and breeding lines (87 accessions) revealed nearly identical values (0.074 versus 0.061) (S4 Table) both of which were lower than the molecular diversity of landraces (0.088). Accessions collected from Abia and Ebonyi states had the same π value (0.065). There was no significant difference in π between root flesh coloration traits of cream, white, and yellow coloration, although these colorations were higher than those of other storage root flesh colours (orange and purple) (S4 Table). In terms of clustering based on the NJ tree, π for Groups 3 and 4 were nearly identical (0.068 and 0.069, respectively) S4 Table). Similar result was found in Group 1 and the mixed group based on population structure. Negative Tajima’s D was consistently recorded across all molecular diversity measurements (S4 Table).

Pairwise Nei’s Gst genetic differentiation for all populations was significantly greater than 0, except for Tillaberi and Dosso states (Table 3). This explained discernible population differentiation, which ranged from 0.025 to 0.086 between the three biological status, from 0.061 to 0.261 between the five sampled collection states, from 0.041 to 0.447 between the five countries considered, and from 0.046 to 0.127 between three African regions (Table 3). The Pairwise Nei’s Gst comparison between the five countries revealed that the highest differentiation was between Niger and Uganda, as well as between Niger and Mozambique, while the smallest difference was registered between Nigeria and Mozambique.

**Table 3:** Pairwise Nei’s genetic differentiation (Gst) analysis at different hierarchical levels using 271 *Ipomoea batatas* (sweetpotato) accessions genotyped with 7,591 polymorphic SNP markers.

a. Population pairwise Nei’s Gst values computed to understand the extent of genetic differentiation (divergence) among the three biological statues						
**Biological statues**	**Improved line**	**Landrace**	**Breeding line**	** **	** **	** **
**Improved line**	0					
**Landrace**	0.045	0				
Breeding **line**	0.025	0.086	0			
b. Population pairwise Nei’s Gst values computed to understand the extent of genetic differentiation (divergence) among the five collecting state						
**States of collection**	**Abia**	**Ebonyi**	**Dosso**	**Tillaberi**	**Atlantique**	** **
**Abia**	0					
**Ebonyi**	0.061	0				
**Dosso**	0.177	0.131	0			
**Tillaberi**	0.261	0.214	-0.115	0		
**Atlantique**	0.088	0.08	0.085	0.166	0	
c. Population pairwise Nei’s Gst values computed to understand the extent of genetic differentiation (divergence) among the 5 countries						
**Countries**	**Nigeria**	**Uganda**	**Mozambique**	**Niger**	**Benin**	** **
**Nigeria**	0					
**Uganda**	0.13	0				
**Mozambique**	0.041	0.127	0			
**Niger**	0.251	0.447	0.301	0		
**Benin**	0.08	0.127	0.09	0.18	0	
d. Population pairwise Nei’s Gst values computed to understand the extent of genetic differentiation (divergence) between three Africa regions						
**Africa regions**	**West Africa**	**East Africa**	**South Africa**	** **	** **	** **
**West Africa**	0					
**East Africa**	0.103	0				
**South Africa**	0.046	0.127	0			
e. Population pairwise Nei’s Gst values computed to understand the extent of genetic differentiation (divergence) among the four groups based on cluster analysis						
**Groups based on cluster analysis**	**G1**	**G2**	**G4**	**G3**		
**G1**	0					
**G2**	0.152	0				
**G4**	0.223	0.122	0			
**G3**	0.254	0.152	0.165	0		
f. Population pairwise Nei’s Gst values computed to understand the extent of genetic differentiation (divergence) between the different flesh colour						
**Flesh colours**	**White**	**Purple**	**Orange**	**Yellow**	**Cream**	**Unknow**
**White**	0					
**Purple**	0.155	0				
**Orange**	0.078	0.109	0			
**Yellow**	0.022	0.144	0.059	0		
**Cream**	0.014	0.171	0.079	0.03	0	
**Unknow**	0.006	0.168	0.0838	0.021	0.008	0
g. Population pairwise Nei’s Gst values computed to understand the extent of genetic differentiation (divergence) among the different population structure at K = 4						
**Groups based on population structure**	**Mixed**	**G2**	**G1**	**G4**	**G3**	
**Mixed**	0					
**G2**	0.143	0				
**G1**	0.036	0.182	0			
**G4**	0.223	0.428	0.25	0		
**G3**	0.168	0.391	0.197	0.412	0	

Based on biological status, breeding lines and landraces showed the highest value of pairwise Nei’s Gst, while the lowest was between breeding lines and improved lines. The highest pairwise Nei’s genetic differentiation (Gst) was observed between the purple storage root flesh colour and other colour groups, with values exceeding 0.10. This Gst value gradually decreased for other colour groups, such as cream (0.117), white (0.115), yellow (0.144), and orange (0.109). Based on colour, the lowest pairwise Nei’s Gst values were registered between white and cream (0.014), and between white and yellow (0.022).

Pairwise predictions derived from cluster analysis were highest between group 1 and group 3 (0.254), the lowest between group 2 and group 3 (0.122). Based on population structure, group 2 and group 4 had the highest difference (0.428), whereas group 1 and group 2 were the most closely related.

### Gene flow and genetic identity

The gene flow (Nm) estimates across different hierarchical levels are presented in Table 4. Gene flow for biological statues varied from 1.381 to 7.021, which were high according to the interpretation guidelines [37, 38, 53, 54]. The state of accession collection, indicated that the highest gene flow (24.848) was computed between Dosso (Niger) and Atlantique (Benin), while the lowest gene flow (0.615) was observed between Abia (Nigeria) and Tillaberi (Niger). Sweetpotato accessions originating from Nigeria and Mozambique exhibited the highest gene flow value (5.876). When the four groups based on cluster analysis and population structure at K = 4 were considered, gene flow varied from 0.397 to 0.917 and 0.179 to 0.677, respectively, which were low based on the interpretation guidelines [37, 38, 53, 54]. The gene flow from white fleshed to cream fleshed sweetpotato was the highest (14.213), followed by gene flow from white fleshed to yellow fleshed sweetpotato (7.830). The lowest gene flow was from purple fleshed to cream fleshed sweetpotato genotypes (0.815).

**Table 4:** Pairwise estimates of gene flow (Nm) for different hierarchical levels using 271 *Ipomoea batatas* accessions genotyped with 7,591 polymorphic SNPs.

a. Pairwise estimates of gene flow (Nm) values computed to understand the extent of genetic differentiation (divergence) among the three biological statues					
	**Improved line**	**Landrace**	**Breeding line**		
**Improved line**	0				
**Landrace**	3.239	0			
**Breeding line**	7.021	1.381	0		
b Pairwise estimates of gene flow (Nm) values computed to understand the extent of genetic differentiation (divergence) among the five collecting state					
	**Abia**	**Ebonyi**	**Dosso**	**Tillaberi**	**Atlantique**
**Abia**	0				
**Ebonyi**	2.151	0			
**Dosso**	1.251	3.819	0		
**Tillaberi**	0.615	0.882	-5.152		
**Atlantique**	1.315	1.571	24.848	1.337	0
c. Pairwise estimates of gene flow (Nm) values computed to understand the extent of genetic differentiation (divergence) among the 5 countries					
	**Nigeria**	**Uganda**	**Mozambique**	**Niger**	**Benin**
**Nigeria**	0				
**Uganda**	1.923	0			
**Mozambique**	5.876	2.97	0		
**Niger**	0.641	0.156	0.398	0	
**Benin**	1.466	1.862	1.63	1.072	0
d. Pairwise estimates of gene flow (Nm) values computed to understand the extent of genetic differentiation (divergence) among the four groups based on cluster analysis					
	**G1**	**G2**	**G4**	**G3**	
**G1**	0				
**G2**	0.782	0			
**G4**	0.492	0.917	0		
**G3**	0.397	0.713	0.65	0	
e. Pairwise estimates of gene flow (Nm) values computed to understand the extent of genetic differentiation (divergence) among the different flesh colours					
	**White**	**Purple**	**Orange**	**Yellow**	**Cream**
**White**	0				
**Purple**	0.975	0			
**Orange**	1.515	1.613	0		
**Yellow**	7.83	1.092	2.153	0	
**Cream**	14.213	0.815	1.531	5.808	0
f. Pairwise estimates of gene flow (Nm) values computed to understand the extent of genetic differentiation (divergence) among the different population structure at K = 4					
	**Mixed**	**G2**	**G1**	**G4**	**G3**
**Mixed**	0				
**G2**	0.874	0			
**G1**	3.664	0.677	0		
**G4**	0.47	0.168	0.433	0	
**G3**	0.675	0.199	0.577	0.179	0

The estimates of genetic identity across various hierarchical levels are shown in Table 5. The genetic identity between Improved and Breeding Line populations is the highest at 0.961, while moderate genetic differentiation was observed between Landrace and Breeding Line at 0.899. Abia and Ebonyi exhibit the highest genetic identity of 0.927, whereas the lowest value (0.631) was found between Dosso and Abia. When considering the countries of origin, the highest genetic identity (0.933) was between Nigeria and Mozambique, and the lowest (0.473) between Uganda and Niger. The highest genetic identity was between G1 and G2 (0.864), while moderate genetic differentiation was computed between G1 and G4 (0.780), indicating high genetic similarity between these cluster groups. At K = 4, the genetic identity between G4 and G2 is the lowest at 0.622. White and Cream fleshed colours exhibited the highest genetic identity at 0.976, compared to Purple and Cream fleshed colour groups, which showed the lowest value at 0.792.

**Table 5:** Pairwise estimates of genetic identity (GI) for different hierarchical levels using 271 *Ipomoea batatas* accessions genotyped with 7,591 polymorphic SNPs.

a. Pairwise estimates of genetic identity (GI) values computed to understand the extent of genetic differentiation (divergence) among the three biological statues					
	**Improved**	**Landrace**	**Breeding line**		
**Improved**	0				
**Landrace**	0.936	0			
**Breeding_line**	0.961	0.899	0		
b. Pairwise estimates of genetic identity (GI) values computed to understand the extent of genetic differentiation (divergence) among the five collecting state					
	**Abia**	**Ebonyi**	**Dosso**	**Tillaberi**	**Atlantique**
**Abia**	0				
**Ebonyi**	0.927	0			
**Dosso**	0.631	0.681	0		
**Tillaberi**	0.675	0.73	0.907	0	
Atlantique	0.896	0.903	0.726	0.777	0
c. Pairwise estimates of genetic identity (GI) values computed to understand the extent of genetic differentiation (divergence) among the 5 countries					
	**Nigeria**	**Uganda**	**Mozambique**	**Niger**	**Benin**
**Nigeria**	0				
**Uganda**	0.751	0			
**Mozambique**	0.933	0.747	0		
**Niger**	0.705	0.473	0.656	0	
**Benin**	0.905	0.749	0.878	0.785	0
d. Pairwise estimates of genetic identity (GI) values computed to understand the extent of genetic differentiation (divergence) among the four groups based on cluster analysis					
	**G1**	**G2**	**G4**	**G3**	
**G1**	0				
**G2**	0.864	0			
**G4**	0.78	0.866	0		
**G3**	0.754	0.835	0.82	0	
e. Pairwise estimates of genetic identity (GI) values computed to understand the extent of genetic differentiation (divergence) among the different flesh colours					
	**White**	**Purple**	**Orange**	**Yellow**	**Cream**
**White**	0				
**Purple**	0.811	0			
**Orange**	0.907	0.869	0		
**Yellow**	0.964	0.816	0.922	0	
**Cream**	0.976	0.792	0.903	0.952	0
f. Pairwise estimates of genetic identity (GI) values computed to understand the extent of genetic differentiation (divergence) among the different population structure at K = 4					
	**Mixed**	**G2**	**G1**	**G4**	**G3**
**Mixed**	0				
**G2**	0.867	0			
**G1**	0.955	0.827	0		
**G4**	0.775	0.622	0.742	0	
**G3**	0.836	0.649	0.803	0.632	0

### Genetic relationship and population structure

**Fig 1:**
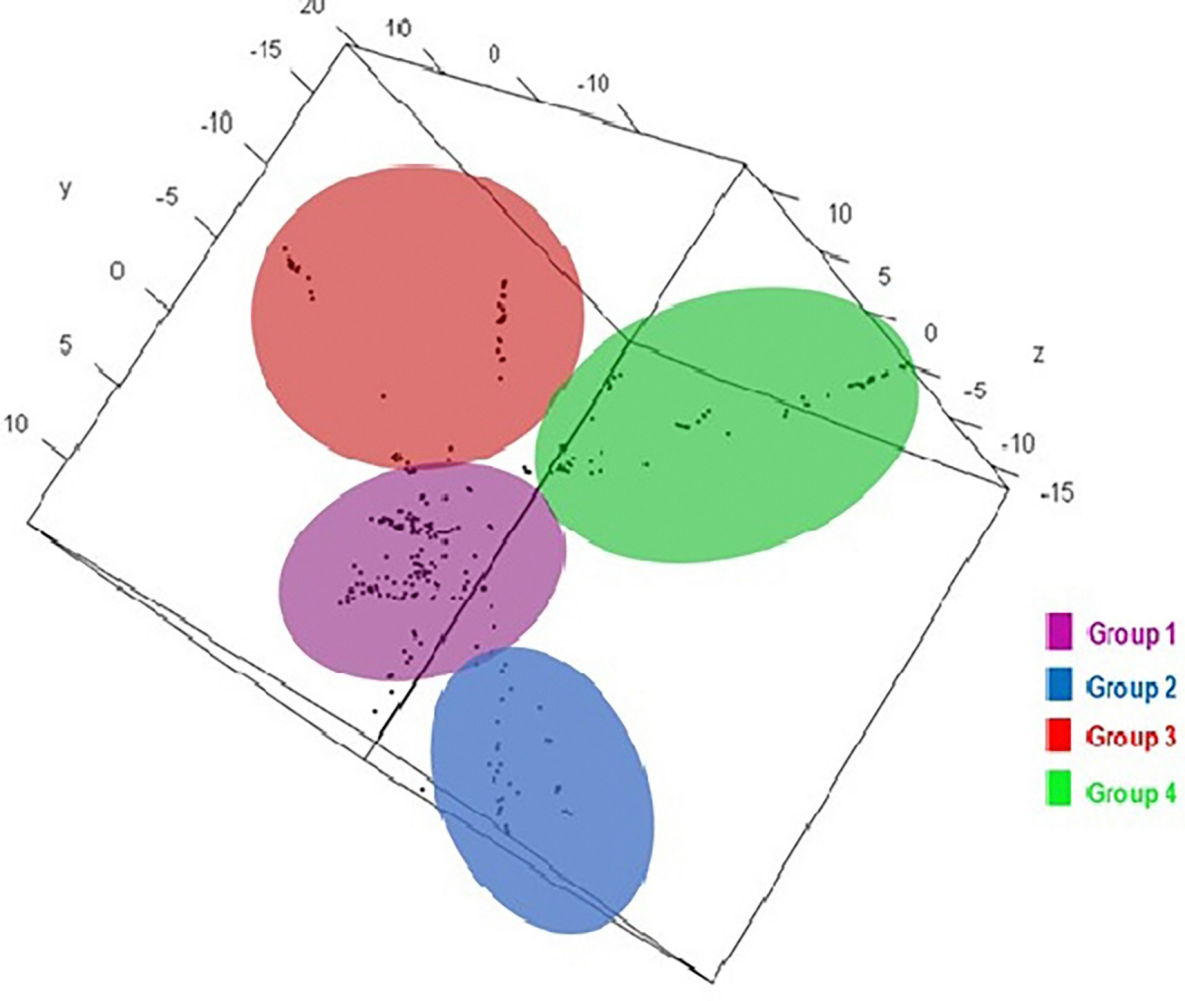
Principal component analysis of 271 *Ipomoea batatas* accessions using 7,591 polymorphic SNPs markers.

**Fig 2:**
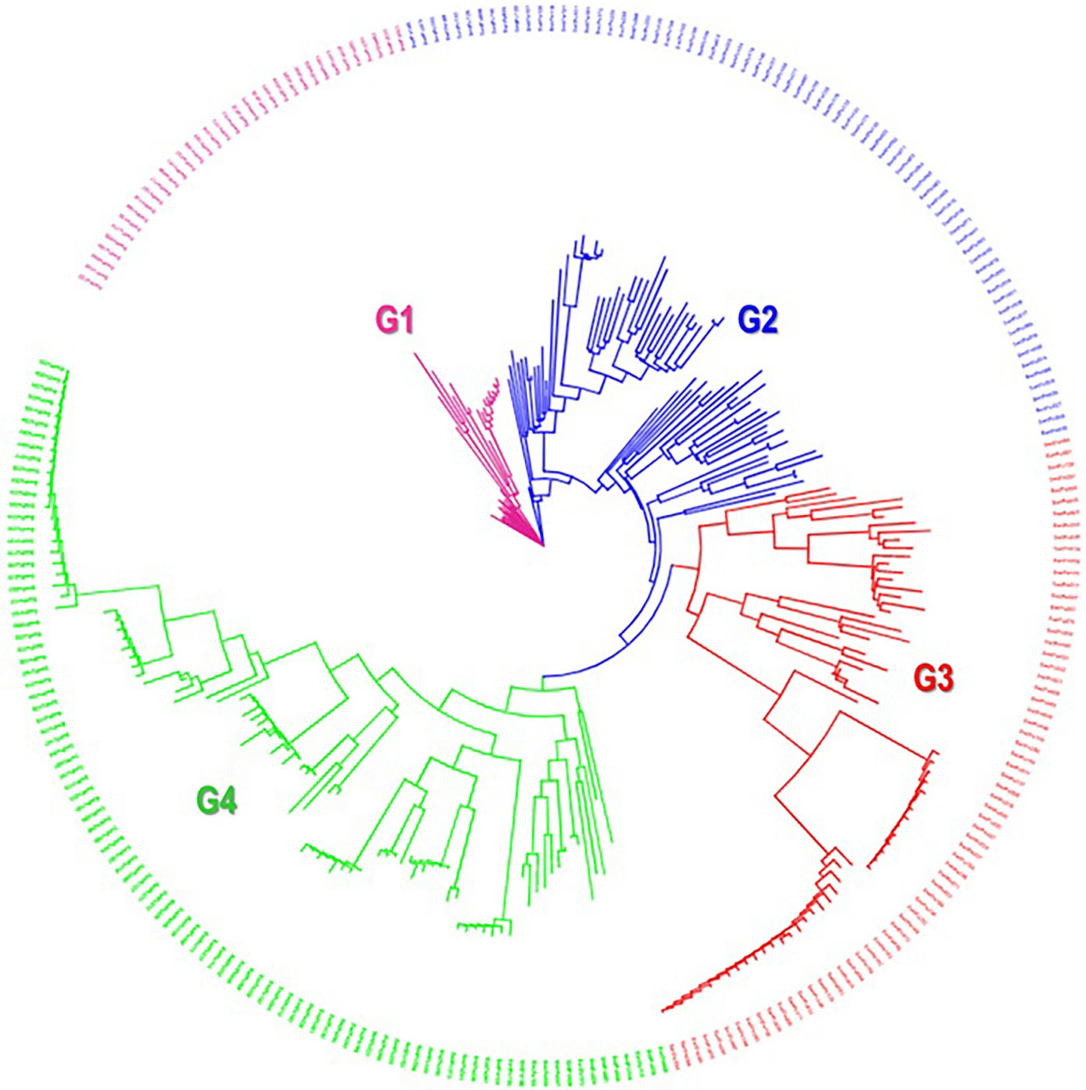
Neighbor-Joining tree based on 7,591 polymorphic SNPs from genotyping by sequencing data for 271 *Ipomoea batatas a*ccessions.

The cumulative variation of the first ten principal components computed across all the 271 accessions explained 46% of the molecular variation (S5 Table). The first, second and third axes explained 12%, 10% and 5% of the overall variance, respectively. G2 was highly and positively correlated with PC2 whereas, G4 was highly and positively correlated with PC1 (Fig 1). However, both G2 and G4 were moderately correlated with PC3. Plotting the accessions onto the first three axes (Fig 1) showed clear population structure similar to the neighbour-joining (NJ) tree (Fig 2). Based on the 7,591 polymorphic SNP markers, the 271 accessions clustered into four groups (Fig 2). The largest group (G4) contained 96 accessions (Fig 2, S1 Table) many of which came from West African regions (94%) and mostly collected from Atlantique site (92%) in Benin (S3, S4 and S6 Figs). This group had a high proportion of white (43%) and yellow (21%) storage root flesh colour accessions (S1 Table; S3 and S7 Figs). While biological status indicated that landrace genotypes were the most common, white and orange sweetpotatoes predominated in this group in terms of storage root flesh colours. The second landrace group (group 3) contained 68 accessions (S1 Table), mostly composed of West African sweetpotato accessions from Benin (53%) and Nigeria (45%) with white (40%) and yellow (20%) storage root flesh colour (S1 Table; S3 and S7 Figs). Most breeding and improved sweetpotato lines with orange storage root flesh coloration (90%) belonged to Group G2, which comprised 72 accessions mostly collected from Abia and Ebonyi states in Nigeria (90%) (S1 Table; S3 and S7 Figs). The last group, Group G1, was composed of 35 accessions, all collected from Abia and Ebonyi states in Nigeria (S6 Fig). The predominant accessions in this group were breeding lines (80%) with orange storage root flesh colour (71%) (S1 Table; S3 and S7 Figs).

**Fig 3:**
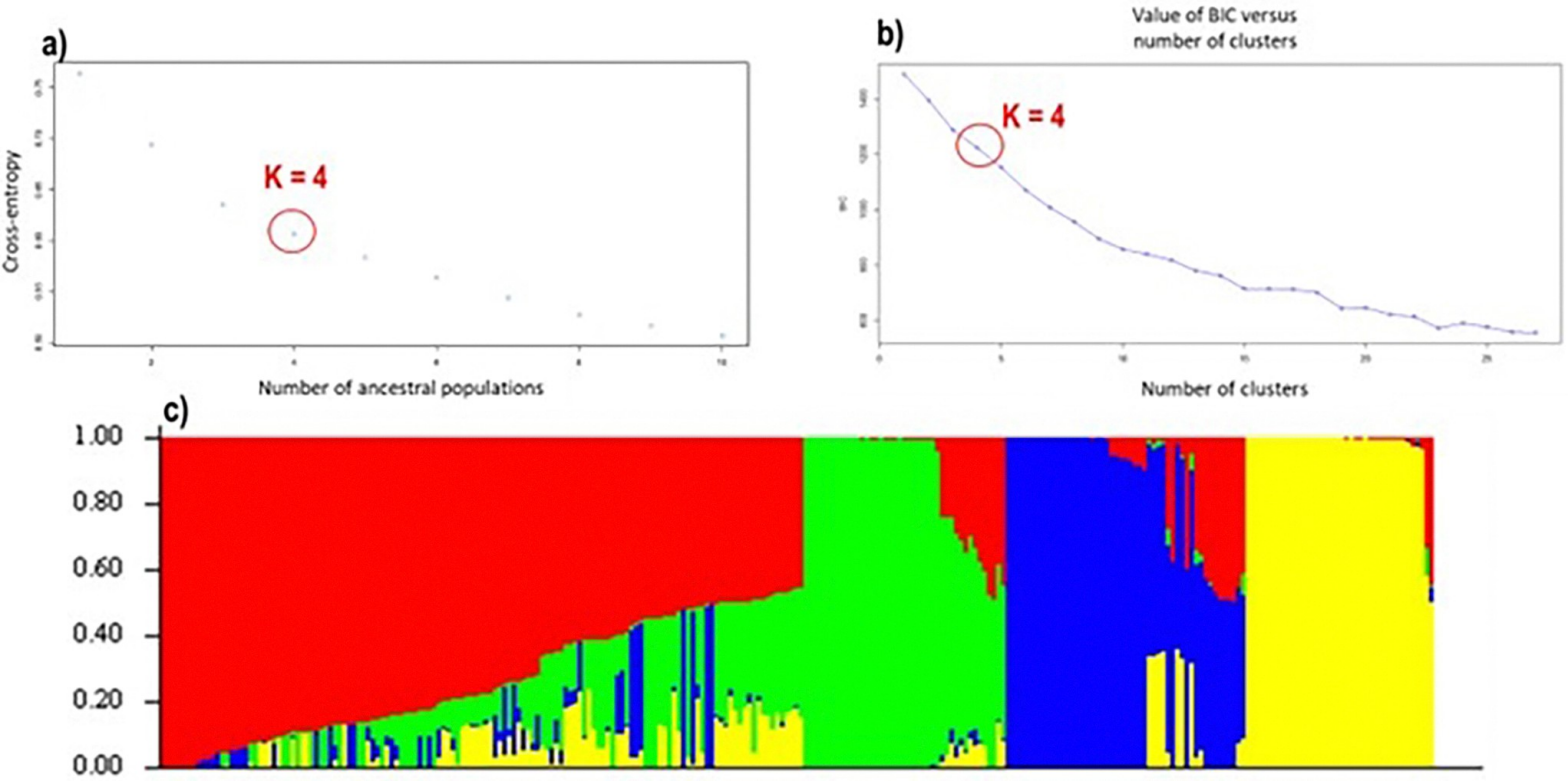
(a) Graph representing cross-entropy vs. number of ancestral populations; (b) Graph representing number of clusters vs. Bayesian Information Criterion (BIC); (c) Population structure with k = 4. The red circle indicates the choice of the number of clusters at k = 4.

Population structure and genetic relationships was evaluated using the STRUCTURE model-based approach [48]. Population structure split the 271 sweetpotato accessions into four groups at K = 4 (Fig 3C, S1 Table). Population 1 had 98 accessions and was composed of a mixture of advanced lines, improved lines, and landraces from all countries considered in this study. Population 2, mostly comprised of Nigerian advanced lines (75.75%), contained 33 accessions. Population 3 contained 39 accessions, exclusively landraces from Benin, and population 4 contained 38 accessions composed of only landraces from West African countries. Additionally, 63 admixed accessions were grouped together with a membership probability less than 0.60 (S1 Table). STRUCTURE analysis closely matched the Principal Component Analysis (PCA) and Neighbor-Joining (NJ) tree clusters based on the biological status of the accessions (S3 Fig). Most accessions in population 2 belonged to the same cluster as those in group G1 (93%). Except for one, all the 38 accessions in population 3 were also found in the G4 cluster. All accessions in population 4 were found in cluster G3. The G2 and G4 accessions made up 78% of population 1.

## Discussion

### Genotyping of West African sweetpotato

A total of 29,523 DArTseq SNP markers were obtained by genotyping 271 sweetpotato accessions collected from West Africa. This number of SNPs was three times greater than that reported by Gemenet et al. [36], who genotyped 662 parents of international potato center (CIP) breeding population using DArTseq tools (29,523 SNPs versus 9,670 SNPs). Following the methodology of Gemenet and collaborators [36], we filtered the SNPs and retained only 7,591 DArTseq SNP markers (25.71% of 29,523 SNPs), which were highly polymorphic among the 271 genotyped samples. Table 1 showed that the number of filtered SNPs ranged from 344 to 773 per chromosome, which was larger than that reported by Gemenet et al. [36], (6 to 18 per chromosome). This number is sufficient and may be useful for analysing genetic diversity. Nevertheless, the methodology employed in this study demonstrates its capability to generate a robust SNP dataset suitable for detailed genetic studies, surpassing previous efforts in SNP marker density. This dataset has significant potential for enhancing genetic diversity analysis, particularly in sweetpotatoes originating from West African countries.

### Genetic diversity and distance

Molecular markers such as SSR markers have been widely used to study genetic diversity of sweetpotato collected from West African countries. However, most studies used a small number of these markers (between 5 and 30) to explored genetic diversity of sweetpotato accessions, resulting in limited insights into genetic variability [55].

For instance, a study of Nigerian sweetpotato cultivars used 5 SSR primers resulted in PIC values ranging only from 0.04 to 0.39 [23]. Comparable findings were reported in Burkina Faso [21], Ghana [13] and Togo [22]. Our present study allowed to explore, for the first time, the genetic diversity of sweetpotato accessions collected from West African countries such as Niger, Nigeria, and Benin using genotyping by sequencing tools (GBS). We calculated Polymorphism Information Content (PIC) and Minor Allele Frequency (MAF) for all SNPs in the genotyped West African sweetpotato accessions and found that the selected SNPs used in this study had a high discriminatory power, as evidenced by the wide range of PIC values (0.25 to 0.5) and MAF values (0 to 0.5). The greatest PIC values previously reported for sweetpotato ranged from 0 to 0.37 [35]. The high PICs (0.25–0.50), MAF (0.10–0.50), and He (0.00–0.80) values in our study were primarily attributable to diversity of accessions owing to the origin of genotypes from different countries, and the large number of polymorphic SNPs. Therefore, West African sweetpotato breeders may find these accessions useful as a source of genetic variation to develop new cultivars.

Our results also demonstrated the presence of a wide variation in genetic distances between sweetpotato accessions, with 12.82% of the pairings being weakly distant, 80.78% of the pairs being moderately distant, and 6.41% of the pairs being highly distant. This further suggested that the sweetpotato accessions included in this study had a low percentage of redundant accessions that contributed very little to the observed genetic variation and genetic divergence. Abundant genetic variability can strengthen the ability of a species to respond to changing environments and consequently enhance its evolutionary potential [56]. Genetic diversity is affected by many factors such as breeding system, seed dispersal, life form, geographical distribution, and historical origin [56]. The germplasm that was analysed in this study contains highly variable genetic material to be able to withstand the aforementioned factors and to be able to support the breeding of new sweetpotato cultivars. However, additional screening could help to identify potential donor parents for the development of new sweetpotato varieties that are resistant or tolerant to biotic and/or abiotic stresses. The high discriminatory power of the selected SNPs used in this study surpasses that of the SSRs previously used in West Africa [23]. While this increase may be significant statistically, inclusion of additional informative SNPs by analysing a large set of collections may be required in the future. Nonetheless, the present study established a new set of markers that were reliably useful for studies of genetic diversity. These markers together with the entire data generated in this study could contribute to the improvement of West African digital sequence information and genetic sequence data.

### Population differentiation and genetic structure

The Analysis of Molecular Variance (AMOVA) based on *PhiPT* values revealed that most of the genetic diversity occurred within populations (74.74 to 94.43%) while the variability among populations ranged from 5.57% to 25.25% (Table 2). These results underscore the genetic richness within West African sweetpotato accessions and also suggested significant evolutionary processes shaping the genetic structure of sweetpotato accessions across different region. *PhiPT analogue of F*_*ST*_ values provided an indication of the evolutionary processes that influence the structure of genetic variation among populations or groups, with values <0.05 which indicated little, 0.05–0.15 moderate, 0.15–0.25 great, and > 0.25 very great genetic differentiation [57]. Great genetic differentiation was found in this study when accessions were clustered using cluster analysis and population STRUCTURE at K = 4 (*PhiPT* = 0.19 and 0.25, respectively). These results suggested that the accessions analysed were better classified using DArTseq SNPs molecular markers and could be used to guide selection of the best parental candidates for specific traits in sweetpotato breeding programmes. The selection could be focused to drought tolerant and resistant to pest and disease parents for development of new farmers varieties [58] since drought stress is a major abiotic constraint and sweetpotato weevil is a major biotic constraint [59]). In comparison to Su et al. [35], the variation between populations observed in all categories in this study was at least three-fold larger than the one reported by Su and collaborators [35]. The percentage of variation among countries of origin (11.42%) was higher than those computed based on States of collection (11.41%), biological status (9.13%), and storage root flesh colour (7.90%), suggesting that genetic flow or migration (emigration or immigration from one geo-graphical area to another) is the most likely evolutionary force acting on this collection [31].

### Molecular diversity indices and genetic differentiation

High values of polymorphic sites (Ps), theta (θ), and nucleotide diversity (π) indicated high genetic variation. Compared to other accessions, landraces had high π (0.088 versus 0.074 and 0.061) (S4 Table) which was to be expected. Landraces have been described as being relatively complex and genetically diverse, and contain several resistance / tolerance genes to biotic and abiotic stresses [60]. Furthermore, Lodhi et al. study [61] previously demonstrated that the landraces have the highest heterozygosity compared to the breeding lines. Our study highlights that West African sweetpotato landraces exhibit higher nucleotide diversity (π), indicating their extensive genetic variability, which aligns with their reputation for genetic complexity and resilience against environmental stresses. Thus, screening or agro-morphological characterisation of these landraces could help to identify potential donor parents for breeding. Highest π obtained from white and yellow colours suggested that variation for this trait exists within each group. Diversity in storage root flesh colour (white and orange) has been reported [13, 62] and different sweetpotato storage root flesh colours have shown their superiority and stability [8]. By selecting various root colour categories that are appropriate for distinct product profiles, breeders could make the best use of this collection. Negative Tajima’s D in this study indicated an excess of rare variation, consistent with population growth or positive selection, highlighting the dynamic genetic landscape of the sweetpotato germplasm under study [63]. This may also suggest that the sweetpotato germplasm now grown in these countries and examined in this study contains an excess of rare alleles, which may be related to previous cycles of selection. High pairwise Nei’s Gst genetic differentiation between accessions from Niger and Mozambique as well as Niger and Uganda could be attributed neither to the distances between the countries, nor the relationship between the two sweetpotato breeding programs. Thus, it is possible that enough time may have elapsed to allow for a higher level of genetic differentiation between accessions of these countries as a result of genetic drift or mutation. These genotypes may be useful for exploring heterosis. However, the smallest Nei’s Gst between Nigeria and Mozambique accessions could be explained by the fact that 51 out of 87 advanced lines from Nigeria were crossed with genotypes from Mozambique. The groups based on advanced lines and landraces had the highest pairwise Nei’s Gst (0.086), while the groupings based on advanced lines and improved lines had the lowest Gst (0.025). A similar result was reported when landraces were analysed and grouped separately from modern varieties [35] and from breeding lines [61]. The results could also have been influenced by storage root flesh colour where 83 out of 87 breeding lines (95.40%) were orange-fleshed storage root, whereas 96 out of 165 landraces (58.18%) were white-fleshed storage root. Using storage root flesh colour, this study found that there is a significant genetic distance between purple and white/cream as well as between orange and white/cream, underscoring their potential utility in breeding programs targeting specific nutritional or industrial traits. Previous research has shown that there was a high diversity in storage root flesh colour [13, 62, 64]. According to Leite et al. [65], the set of purple colour samples exhibited remarkable differences, with the colour range extending from white to yellow, orange, and purple. White and cream fleshed sweetpotato contains high level of dry matter and starch content, while orange-fleshed ones contain high beta-carotene content, and purple types are rich in anthocyanin. Due to the differences in the flesh colour of storage roots, these genotypes could be utilized in various food forms, health improvement, and as parents to breed progenies with high beta-carotene (BC) or high dry matter content (DMC) [64].

### Gene flow and genetic identity

Our study revealed that regions such as Dosso (Niger) and Atlantique (Benin) as having frequent genetic exchanges, while regions like Abia (Nigeria) and Tillaberi (Niger) face significant barriers to gene flow as stated by previous studies [53, 54]. Sweetpotato accessions from Nigeria and Mozambique show high gene flow values (5.876), which suggested similar farming practices or historical exchanges. Cluster analysis and population structure indicated marked genetic differences between groups, with strong genetic connections between white-fleshed and cream-fleshed sweetpotatoes (14.213) and significant differences between purple-fleshed and cream-fleshed genotypes (0.815). Improved and Breeding Line populations have the highest genetic identity (0.961), reflecting a high level of genetic similarity, while Landrace and Breeding Line populations show moderate differences (0.899). Sweetpotatoes with white and cream fleshed colours have the highest genetic identity (0.976), compared to other colour groups like purple and cream, which show more genetic variation (0.792).

### Genetic relationship and population structure

The first, second, and third axes in PCA explained 12%, 10%, and 5% of the overall variance, respectively. These results align with the clustering observed in the neighbor-joining (NJ) tree, reinforcing the robustness of our findings Figs 1 and 2. These groupings were comparable to those previously reported by Su et al [35]. Almost all of the populations could be clearly separated on the scatter plot. However, the pattern that was obtained from the principal component analysis (PCA) and the neighbor-joining (NJ) tree did not correspond with the geographical origin of germplasm, as previously reported [66]. This discrepancy suggested a high level of genetic exchange and migration among sweetpotato populations across the different regions included in this study. The largest group (G4) had 96 accessions and among the countries in this study, Nigeria and Benin accounted for most of the accessions. Group 4 was mostly composed of landraces (95%). According to a previous study based on phylogenetic tree and unweighted pair-group method with arithmetic mean (UPGMA) the clustering of landraces was found to be separated from modern sweetpotato cultivars [35]. This was in agreement with the loss of diversity associated to breeding. Our study demonstrated that white and orange fleshed sweetpotatoes were the most common types of storage root flesh colours. Previous research has demonstrated that there is genetic diversity in storage root flesh colours [13, 62]. It is possible that the high number of storage root orange-fleshed sweetpotatoes as well as high proportion of orange-fleshed sweetpotatoes in Nigeria and Mozambique accessions could be explained by the fact that both countries have sweetpotato breeding programmes [67], and one of their breeding objectives was to breed sweetpotato cultivar with high beta-carotene content were tightly linked with orange fleshed-colour.

Analysis of population structure split the 271 sweetpotato accessions into four populations based on K = 4, showing congruence with the cluster analysis. In previous studies, 417 sweetpotato genotypes were split into four groups using 43,105 SNPs [68], while 604 sweetpotato accessions from different continents were separated into six groups using 102,870 SNPs [69]. International Potato Centre (CIP) used 20 SSRs markers to separate 5,979 genotypes in to four clusters [61]. This concordance, observed not only with SNPs but also with SSRs, further validated the reliability of our population structure assessment. In this study, accessions with membership probabilities above 60% were assigned to the same group, while those with probabilities below 60% from any group were assigned to a mixed group. The fact that a significant number of accessions were found in population 1 suggests that there is no significant difference between groups, which agrees with [63], but contradicts by Su et al [35]. However, the assignment of improved varieties and breeding lines with 73.68% and 29.88% of the total of each of them, respectively, in population 1 was expected in this study since they were not genetically distant from one another. This was supported by pairwise Nei’s Gst of 0.025, which was the lowest. This low differentiation highlights the success of breeding programs in maintaining genetic similarity among improved varieties. Within populations 2, 3, and 4, the classification of individuals as either landraces or breeding lines/improved varieties was evident. These genotypes may well be used as parents to exploit heterosis by breeding programs in Niger and Nigeria [59, 70]. Both modern varieties and landraces were grouped separately into their own categories, as previously [35], and breeding lines were also separated from landraces [61]; and also, pre-breeding lines were separated from local landraces [71]. The separation of landraces from Benin in population 3 and 4 was unexpected. Two hypotheses can explain this observation: (i) the diverse origins of these accessions and the inability of sweetpotato collection mission to reveal the origin and ancestors of these accessions (ii) the rapid exchange of sweetpotato genotypes between farmers from diverse West African agro-ecologic regions. Genetic flow or migration is considered the most evolutionary factor compared to mutation, selection, genetic drift, and recombination. Most accessions in the STRUCTURE analysis were in the same biological status groups which was consistent with the previous findings [35], but not with the findings of [66]. Each population membership based on population structure was congruent with one group based on cluster analysis. This STRUCTURE based on concordance model with cluster analysis has also been reported using SNPs [35] as well as SSRs for population analysis [72, 73]. However, this concordance did not correspond with the geographical origin of accessions, confirming our previous conclusion based on Principal component analysis (PCA) and Neighbor-Joining (NJ) tree clustering that the sweetpotato accessions included in this study exhibit extensive genetic flow and exchange among regions. Similar findings were previously reported in sweetpotato [35, 68], Taro [33], onion [71] and melon [73] studies. This may suggest that plant genetic resources were rapidly exchanged between regions.

## Conclusion

In this study, 7,591 DArTseq-based SNPs markers were retained after filtering and were highly polymorphic. These SNP markers used revealed substantial variation within and between populations. Notably, variation among countries of origin (11.42%) was higher than that based on biological status (9.13%) and flesh colour of storage roots (7.90%), highlighting the significant impact of migration compared to other evolutionary factors. Population structure based on PCA, NJ tree, and STRUCTURE analysis at K = 4 divided the 271 accessions into four groups. However, this concordance did not correspond with the geographical origin of accessions, which indicated extensive genetic flow and exchange among regions. Group 4, which included 95% landraces, was genetically distant (Nei’s Gst = 0.428) from Group 2, which comprised mostly breeding lines. When considering each of the four groups based on the STRUCTURE analysis at K = 4, the best and most genetically diverse accessions that can be used in the future breeding work were SwtPo045 (landrace from Nigeria), SwtPo031 (breeding line from Nigeria), SwtPo230 (landrace from Benin), and SwtPo216 (landrace from Benin), belonging to G1, G2, G3, and G4, respectively. These groups can serve as two heterotic groups for a heterosis-exploiting breeding scheme (HEBS) or for developing new cultivars through an accelerated breeding scheme (ABS). The genomic profiling of 271 sweetpotato accessions provides a foundation for various studies, such as genome-wide association studies (GWAS) or selection based on genomic-estimated breeding values (GEBV). SNP markers can identify links between molecular markers and traits of interest, such as drought tolerance or pest and disease resistance. Consequently, the potential genes present in these accessions could be utilized to develop genetic markers that facilitate breeding.

## Supporting information

S1 Table Summary of the 271 *Ipomoea batatas* accessions used in the present study, including passport data (germplasm type, ecology, originating country, state of collection, regions in Africa continent, crossing countries, flesh colour); group membership based on different multivariate analyses (clustering, PCA and population structure) and proportion of missing data and observed heterozygosity in all accessions. (XLSX)

S2 Table Summary of the 7,591 polymorphic SNPs used in the present study, including major and minor alleles, allele frequency and chromosomal position of each SNP. (XLSX)

S3 Table Identity-by-state (IBS)-based genetic distance matrix between pairs of 271 accessions using 7,591 polymorphic SNPs. (XLSX)

S4 Table Summary of the proportion of polymorphic sites (Ps), θ, S, π and Tajima’s neutrality test (D) across 271 *Ipomoea batatas* accessions genotyped with 7,591 polymorphic SNPs. (XLSX)

S5 Table Summary of principal component, proportion of total and cumulative variance of the 271 *Ipomoea batatas* accessions used in the present study. (XLSX)

S1 Fig Chromosomal distribution of 7,591 polymorphic SNPs used for genotyping 271 *Ipomoea batatas* accessions, including average map length per SNP (kb). (TIF)

S2 Fig Comparisons of IBS-based genetic distance matrices between 271 *Ipomoea batatas* accessions computed with 7,591 polymorphic SNPs. (TIF)

S3 Fig NJ-tree & PCA between 271 *Ipomoea batatas* accessions computed with 7,591 polymorphic SNPs based on biological status. (PPTX)

S4 Fig NJ-tree & PCA between 271 *Ipomoea batatas* accessions computed with 7,591 polymorphic SNPs based on countries. (PPTX)

S5 Fig NJ-tree & PCA between 271 *Ipomoea batatas* accessions computed with 7,591 polymorphic SNPs based on regions. (PPTX)

S6 Fig NJ-tree & PCA between 271 *Ipomoea batatas* accessions computed with 7,591 polymorphic SNPs based on the state of collection. (PPTX)

S7 Fig NJ-tree & PCA between 271 *Ipomoea batatas* accessions computed with 7,591 polymorphic SNPs based on the flesh color. (PPTX)
