# Supplementary figures and images for: Genetic diversity and population structure studies of West African sweetpotato [Ipomoea batatas (L.) Lam] collection using DArTseq
Source: PLoS One. 2025 Jan 3;20(1):e0312384. doi: 10.1371/journal.pone.0312384 (PMC11698414; doi:10.1371/journal.pone.0312384)

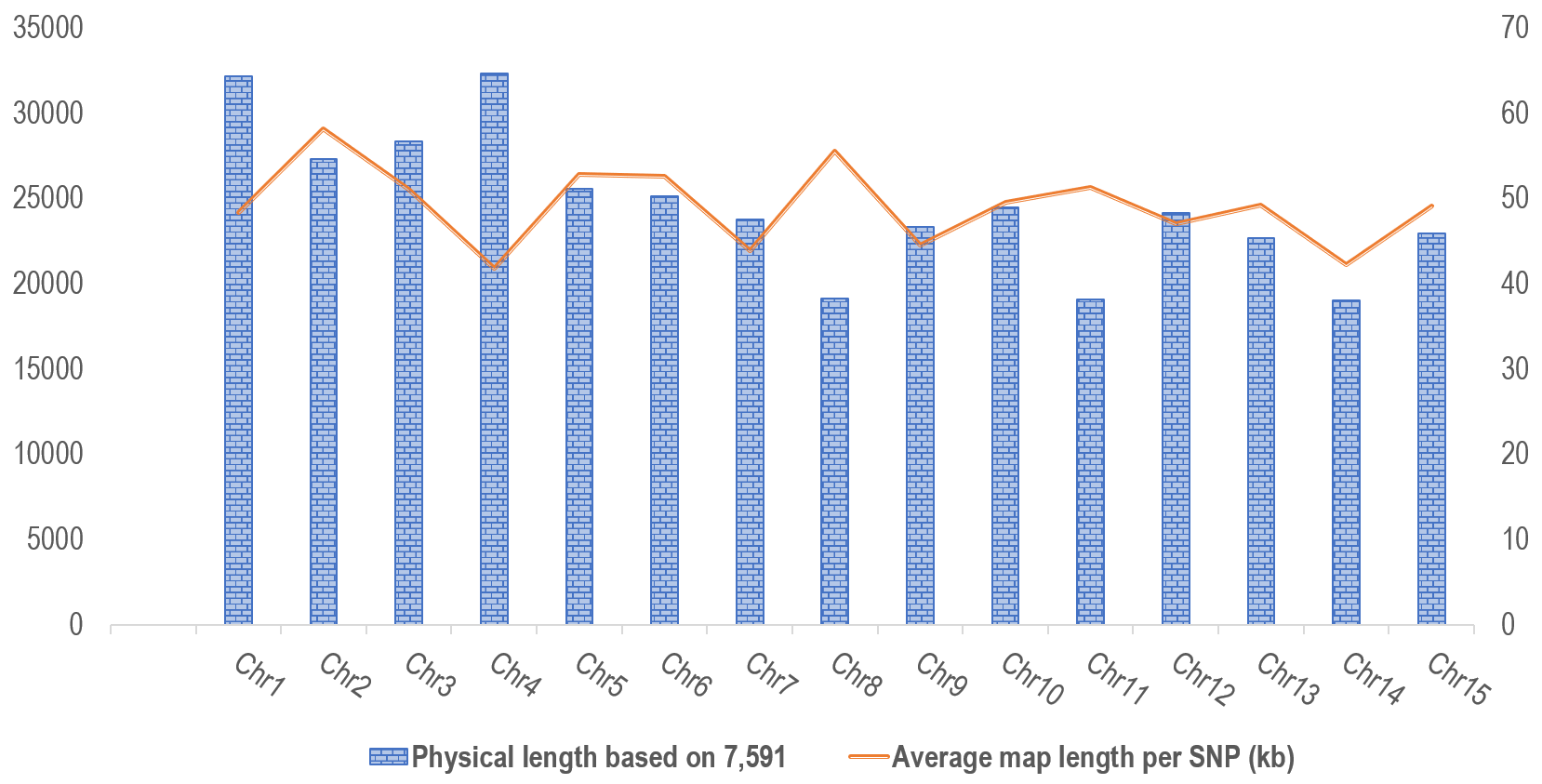

Supplement: S1 Fig — (TIF) [file pone.0312384.s006.tif]

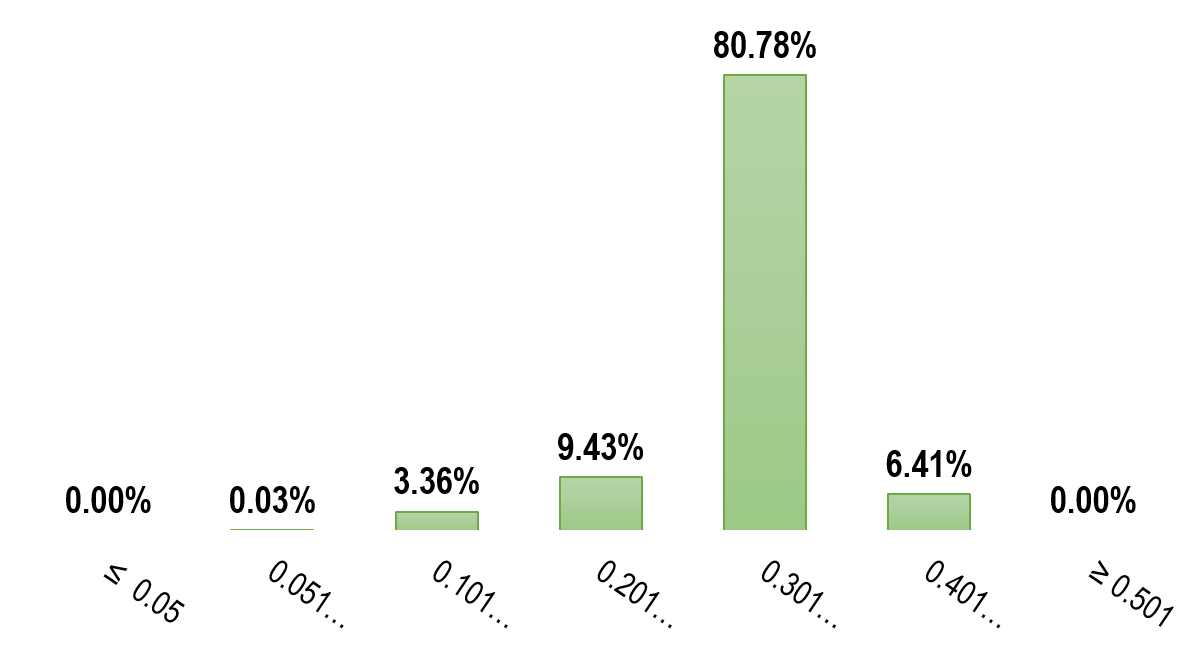

Supplement: S2 Fig — (TIF) [file pone.0312384.s007.tif]
