# Supplementary material for: Genetic diversity and population structure studies of West African sweetpotato [Ipomoea batatas (L.) Lam] collection using DArTseq
Source: PLoS One. 2025 Jan 3;20(1):e0312384. doi: 10.1371/journal.pone.0312384 (PMC11698414; doi:10.1371/journal.pone.0312384)
Supplement: S4 Fig — (PPTX) [file pone.0312384.s009.pptx]

## Slide 1
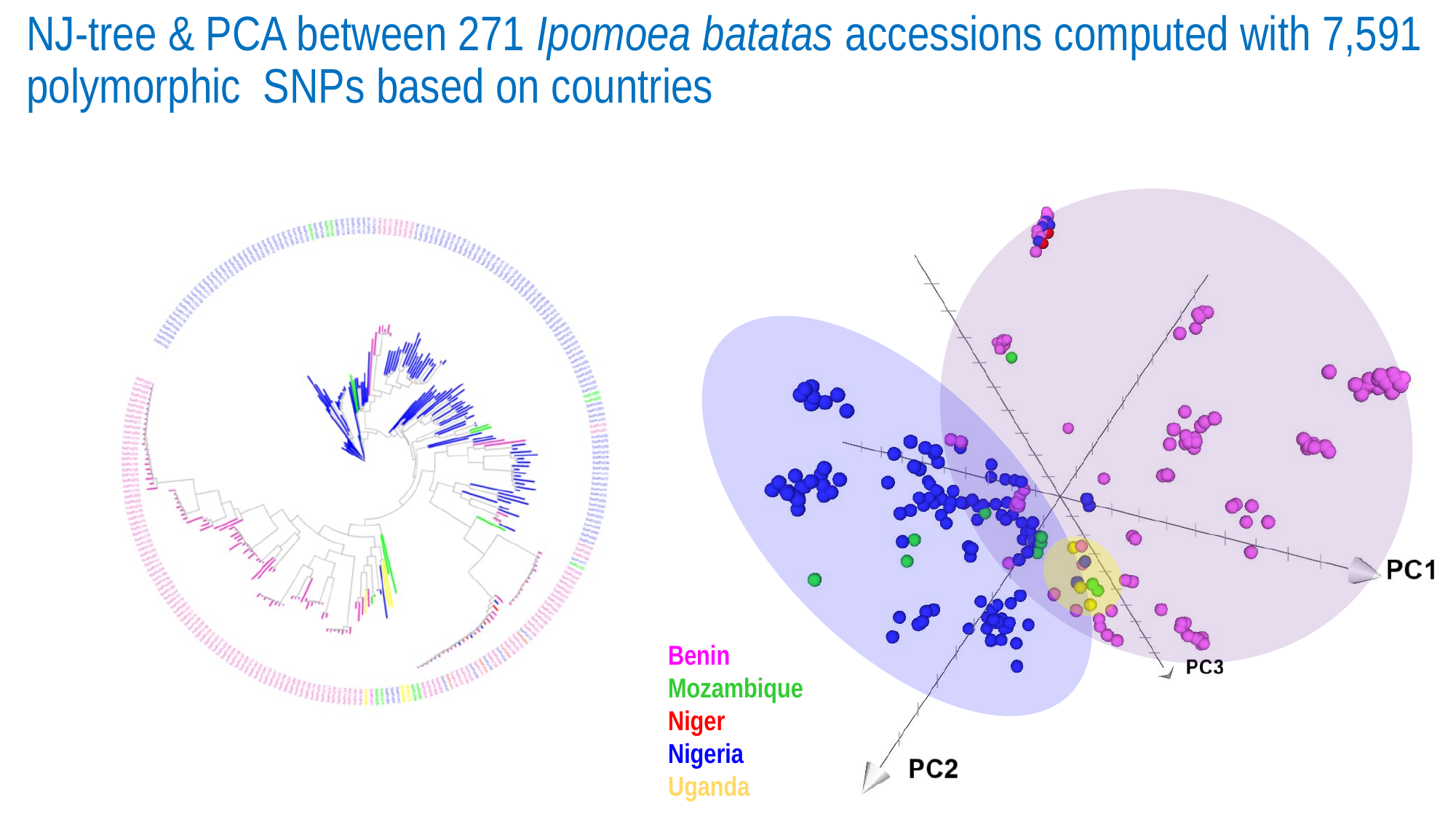

NJ-tree & PCA between 271 Ipomoea batatas accessions computed with 7,591 polymorphic SNPs based on countries
Benin
Mozambique
Niger
Nigeria
Uganda
